# A cross-sectional study of gender differences in quality of life domains in patients with neurofibromatosis type 1

**DOI:** 10.1186/s13023-022-02195-y

**Published:** 2022-02-08

**Authors:** G. Hamoy-Jimenez, H. A. Elahmar, M. Mendoza, R. H. Kim, V. Bril, C. Barnett

**Affiliations:** 1 Elisabeth Raab Neurofibromatosis Clinic, University Health Network, 200 Elizabeth ST, 5EC Room 334, Toronto, ON M5G 2C4 Canada; 2 U-Turn Medical Clinic, Kuwait City, Kuwait; 3 Princess Margaret Cancer Centre, University Health Network, Toronto, Canada; 4 Department of Medicine, University of Toronto, Toronto, Canada; 5 Institute of Health Policy, Management and Evaluation, University of Toronto, Toronto, Canada

**Keywords:** NF1, Gender differences, Perceived physical appearance, Quality of life

## Abstract

**Background:**

There is limited data regarding gender differences in quality of life between women and men with Neurofibromatosis type 1. We aimed to study differences in quality of life domains between women and men with Neurofibromatosis type 1 living in Canada.

**Methods:**

This is a cross sectional study of adults with Neurofibromatosis type 1 attending a tertiary NF centre at Toronto General Hospital between January 2016 to December 2017. Demographic and clinical data were collected. We compared scores of generic measures (SF-36, EQ-5D-5L, pain interference) and a disease-specific measure (PedsQL-NF1 module) between women and men. We also assessed the relationship between disease visibility scored by an examiner (Ablon’s visibility index) and self-reported perceived physical appearance, stratified by gender.

**Results:**

One hundred and sixty-two participants were enrolled, 92 females and 70 males. Ablon’s index score 1 was in 43% and score 2 in 44%, while only 13% of patients had a score 3. Women had worse scores on the total PedsQL-NF1 scales, and also in the perceived physical appearance, anxiety and emotional health domains. In women, there was a low but significant correlation between Ablon’s index and perceived physical appearance (r = − 0.27, p = 0.01, ANOVA p < 0.001). In men, there was no difference in self-reported physical appearance by Ablon’s index. There were no differences between men and women in the SF-36 or EQ-5D-5L scores.

**Conclusion:**

Women with NF1 reported worse NF1-related quality of life than men, with worse perceived physical appearance, anxiety, and mental health. Perceived physical appearance does not always correlate to disease visibility; therefore, healthcare providers should inquire about body image, physical appearance concerns, and mental health, especially among women with NF1.

## Background

Neurofibromatosis type 1 (NF1) is a rare genetic disorder with an autosomal dominant inheritance and an estimated incidence of 1:2500–3000 live births [1–3].

Individuals with NF1 can have different manifestations including cutaneous and plexiform neurofibromas which may cause disfigurement. In addition, they have a higher risk of malignancy and cognitive impairments. Despite some typical manifestations of NF1, there is marked heterogeneity in the NF1-related symptoms among affected persons. The life expectancy of people with NF1 was been estimated to be reduced by 10 years as compared to the general population [4].

The quality of life (QoL) of people with NF1 is consistently below that of the general population and is different from that of patients with other clinical conditions [5–7]. Specific NF1 manifestations that have been linked to reduced QoL are plexiform neurofibroma and associated pain, scoliosis and other visible and potentially disfiguring manifestations of NF1 [8–11].

There is conflicting data in the literature regarding gender differences in the QoL in people with NF1, with some studies reporting no differences and others reporting worse QoL in women [12, 13]. We aimed to assess gender differences in QoL between women and men attending a tertiary NF centre in Toronto, Canada. We hypothesized that women with NF1 would have lower quality of life, mostly driven by disease visibility.

## Methods

This is a secondary analysis of a previously published, cross-sectional study of quality of life of people with NF1 living in Canada [14]. For the original study, we invited individuals meeting criteria for NF1 attending the adult multidisciplinary NF1 clinic at Toronto General Hospital between January 2016 to December 2017 to participate [15]. We collected demographic and clinical data including age, gender, educational attainment, employment status, known plexiform neurofibromas, presence of spinal and brain tumours, other malignancies and history of malignant peripheral nerve sheath tumour (MPNST). Disease visibility was rated by the physician using the Ablon visibility index [16]. Clinicians rated the appearance of NF 1 patients while fully dressed from mild (grade 1) to severe (grade 3). Each NF1 patient completed the following generic and disease specific questionnaires:

1. SF-36: a 36-item scale is a generic QoL measure [17]. The SF-36 assesses eight dimensions, and also has two summary scores for overall physical and mental health. Lower scores indicate worse QoL.
2. The EQ-5D-5L is a preference-based measure consisting of 5 dimensions: mobility (MO), self-care (SC), usual activities (UA), pain/discomfort (PD), and anxiety/depression (AD) [18]. It is scored as health utilities with 0 = death, 1 = perfect health and negative values for disease states valued as worse than death. Additionally, a general health VAS is included, where scores range between 0 (worst possible health) to 100 (best possible health). We used a Canadian valuation algorithm to obtain health utility scores [19].
3. PROMIS pain interference short-form 8a [20]. It is a measure of the degree to which chronic pain interferes with activities of daily living. Higher scores indicate worse pain interference.
4. Peds QL adult NF1 module: a 74-item questionnaire specifically developed for NF1 [21]. It evaluates 16 domains: physical, emotional, social, and cognitive functioning, as well as communication, worry, perceived physical appearance, pain and hurt, paraesthesia, skin irritation, sensation, movement and balance, daily activities, fatigue, treatment anxiety and sexual functioning. Each item is scored in a 5-option Likert scale ranging from 0—never, to 4—almost always. Items are reverse scored and linearly transformed to 0 to 100. Higher scores indicate better QoL.

Analyses: Continuous data are presented as means ± SD, and categorical data as number and %. We compared the mean scores between women and men in different PROs, using t-tests. We also compared mean SF-36 scores of women and men to Canadian normative data stratified by gender, using Bonferroni correction for multiple testing [22]. We used chi-squared test to compare proportions. To assess the effects of physical appearance, we compared Ablon’s visibility index—completed by an examiner—between men and women. To assess the patients’ assessment of their disease visibility, we used the perceived physical appearance score of the Peds-QL NF1 module. We assessed the relationship between disease visibility as observed by a rater (Ablon’s) to the patient’s perception of skin visibility using ANOVA to compare mean perceived physical appearance scores by Ablon’s class in all patients and stratified by gender.

We also fit a linear regression model to assess the variables associated with perceived physical appearance scores. We included in the model age, gender, presence of a plexiform neurofibroma, spinal tumour, optic glioma, Ablon’s index, educational attainment, marital and employment status.

All statistical analyses were done using R version 4.1.0 [23].

## Results

A total of 162 NF1 patients were enrolled in our study, 92 (57%) females and 70 (43%) males. There were no significant differences in demographic and clinical characteristics between men and women, as seen in Table 1.

**Table1:** Demographics and clinical characteristics

	Men (n = 70) Mean ± SD, or n (%)	Women (n = 92) Mean ± SD, or n (%)	p value
Age (years)	35 ± 15	32 ± 12	0.16
*Marital status*			
Single	46 (66%)	55 (60%)	0.53
Married	17 (24%)	30 (31%)	
Separated	1 (1%)	2 (2%)	
*Education*			
Primary	3 (4%)	2 (2%)	0.33
Secondary	17 (17%)	17 (18%)	
Post-secondary	44 (63%)	71 (77%)	
*Employment status*			
Employed	33 (47%)	42 (46%)	0.69
Not employed	1 (1%)	4 (4%)	
Student	15 (21%)	20 (21%)	
Disability	8 (11%)	16 (17%)	
Homemaker	1 (1%)	3 (3%)	
*Clinical characteristics*			
Ablon’s Visibility Index	1.74 ± 0.73	1.67 ± 0.66	0.59
Brain tumor	19 (27%)	22 (24%)	0.90
Spinal tumor	22 (31%)	20 (21%)	0.89
History of MPNST	6 (8%)	8 (9%)	1.00
Optic glioma	9 (13%)	16 (17%)	0.52
Known plexiform neurofibroma	22 (31%)	41 (45%)	0.08
Other cancers	5 (7%)	9 (10%)	0.63

When using the Ablon’s index as a measure of disease visibility, 43% of patients scored 1, 44% scored 2 and only 13% of patients scored 3. There were no significant differences between men and women on distribution of Ablon scores (Table 1).

**Fig. 1:**
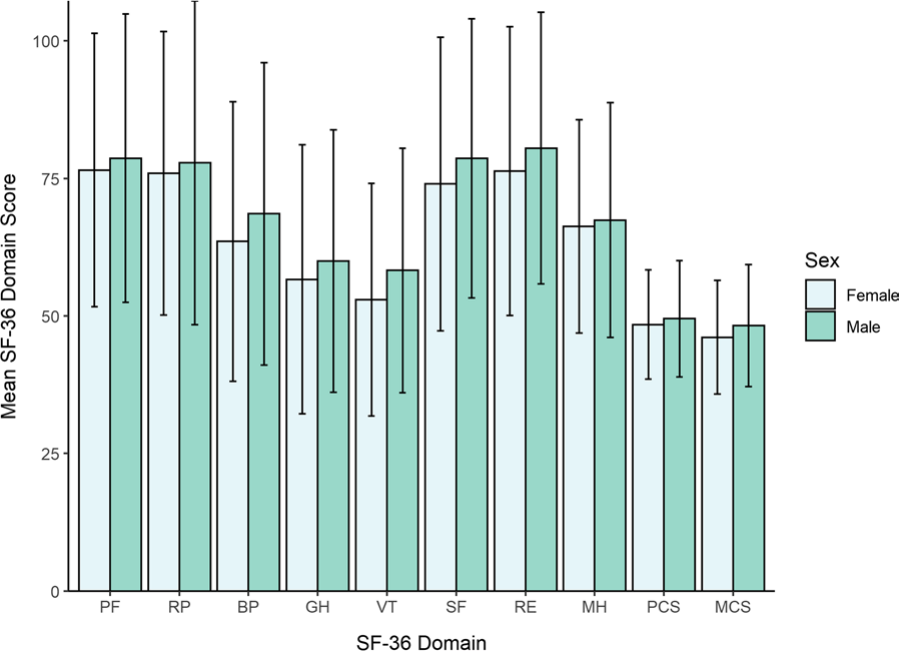
Mean SF-36 scores by gender. SF-36 scales: physical functioning (PF), role physical (RP), bodily pain (BP), general health (GH), vitality (VT), social functioning (SF), role emotional (RE) and mental health (MH). Physical Component Summary (PCS) and Mental Component Summary (MCS). There were no significant differences between women and men (t-test p > 0.05 for all)

There were no significant differences in any of the SF-36 domains or sum-scores between women and men (Fig. 1). When comparing to Canadian normative data, both women and men with NF1 had significantly lower scores than the general population in all domains of SF-36, except for role emotional and the mental component scale, which did not reach significance. The mean EQ-5D-5L utility score was similar between women and men (0.72 ± 0.25 and 0.75 ± 0.24, p = 0.41), as was the global health VAS score (72.4 ± 25.5 for women and 76.9 ± 20.2 for men, p = 0.24). Women had similar scores than men in the PROMIS pain interference scale (52.7 ± 10.3 and 50.3 ± 9.9 respectively, p = 0.87).

When comparing the Peds-QL NF1 module scores, women had significantly lower total scores, indicating worse QoL; they also had lower scores in the emotion, perceived physical appearance and anxiety domains; the most significant difference was in the perceived physical appearance domain (41.4 ± 31.2 and 64.3 ± 30.6, p < 0.0001, Table 2).

**Table 2:** Peds QoL NF1 module scores in men and women

Domains	Women (n = 92) Mean ± SD	Men (n = 70) Mean ± SD	p value	Adjusted p value^§^
Physical function	63.1 ± 29.9	70.6 ± 29.6	0.13	0.22
Emotion	52.5 ± 25.7	64.4 ± 24.5	0.0026	**0.02**
Social	62.8 ± 28.9	67.9 ± 30.1	0.30	0.39
Cognitive	54.7 ± 26.7	59.7 ± 26.0	0.24	0.37
Communication	64.1 ± 29.1	70.6 ± 29.5	0.50	0.61
Perceived physical appearance	41.4 ± 31.2	64.3 ± 30.6	< 0.0001	**0.00017**
Worry	49.6 ± 26.2	58.1 ± 26.6	0.05	0.10
Pain and hurt	55.2 ± 31.9	65.8 ± 32.5	0.048	0.11
Paresthesia	69.8 ± 28.5	75.2 ± 30.7	0.27	0.38
Skin irritation	73.6 ± 20.9	79.7 ± 24.9	0.11	0.20
Sensation	76.5 ± 24.2	84.9 ± 18.6	0.018	0.06
Movement and balance	79.1 ± 22.8	77.1 ± 29.0	0.65	0.73
ADLs	91.6 ± 15.0	92.2 ± 13.6	0.78	0.82
Fatigue	55.1 ± 27.1	64.9 ± 28.1	0.035	0.09
Anxiety	74.1 ± 27.6	85.9 ± 24.6	0.007	**0.029**
Sexual functioning	84.7 ± 20.0	83.4 ± 27.1	0.83	0.83
Total score	64.9 ± 16.5	72.9 ± 18.6	0.006	**0.029**

^§^p value adjusted for multiple comparisons using the Benjamini–Hochberg method and a false discovery rate of 0.05. Bolded values are significant after adjustment

**Fig. 2:**
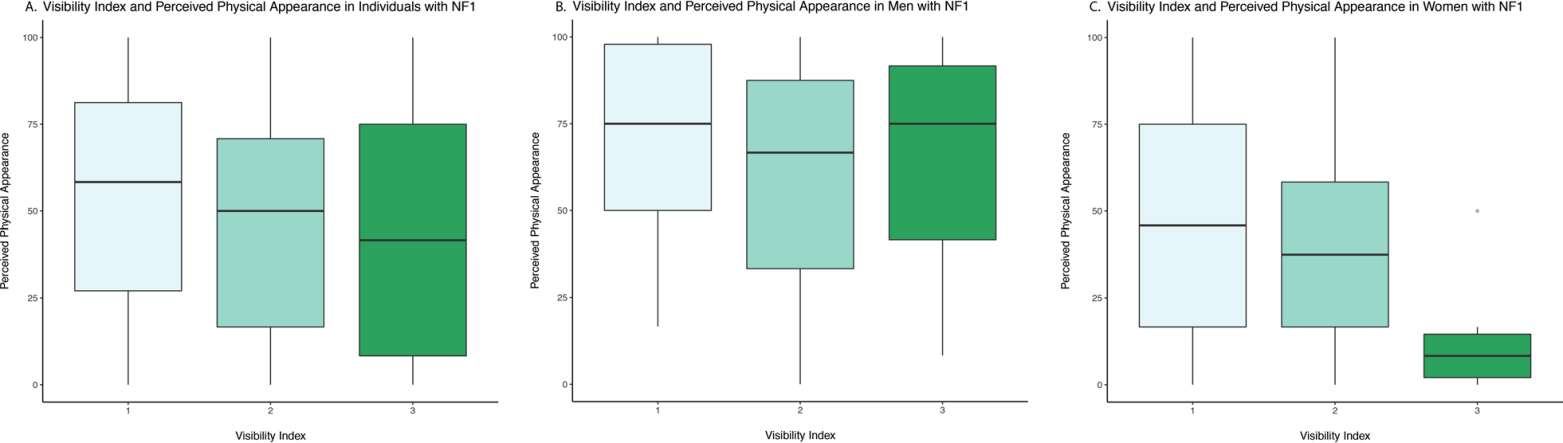
Relationship between perceived physical appearance, disease visibility and gender. These figures depict the relationship between Ablon’s score completed by an examiner, and perceived physical appearance scores (self-reported by patients). Panel A is for all patients, panel B in men and panel C in women. Only in women there was a significant correlation (ANOVA p < 0.0001)

We compared the mean scores on the perceived physical appearance domain by Ablon’s class. When looking at the total cohort (men and women) we found no significant difference. However, when stratified by gender, we found that women had a low, but significant, association between Ablon’s index and perceived physical appearance, with lower scores with increments in Ablon’s class (r = − 0.27, p = 0.01; ANOVA p < 0.001). In men, there was no correlation between self-reported physical appearance by Ablon’s index (r = − 0.08, p = 0.54, Fig. 2).

A linear regression model showed that the main drivers of worse perceived physical appearance were being female and having a plexiform neurofibroma. Ablon’s index barely reached significance (Table 3).

**Table 3:** Regression model for perceived physical appearance

Variable	Estimate	SE	p value
Intercept	66.6	23.1	0.005*
Ablon’s index	− 10.6	5.3	0.048*
Female	− 24.2	7.6	0.002*
Plexiform neurofibroma	− 16.8	7.4	0.026*
Married/partner	30.7	21.7	0.16
Single	24.3	20.4	0.24
Trade or community school	3.27	10.5	0.76
University education	− 10.6	11.5	0.36
Unemployed	13.3	15.7	0.40
Employed	0.7	12.3	0.95
Other Employment	12.4	12.4	0.32
Spine tumour	− 6.6	7.6	0.39
Optic glioma	− 3.0	9.2	0.74

R2: 0.32 p = 0.001

Other employment: student, retired. Employment status compared to being on disability benefits

## Discussion

In this study, we found that women with NF1 reported worse perceived physical appearance than men, while also experiencing worse scores in NF1-related QoL, anxiety and emotional health. This should not be surprising, as studies in the general population have shown that appearance concerns are more common in women than men (61% vs 35%) [24]. This likely reflects the societal expectations around physical appearance that are stronger for women than men [25].

In our study, men had similar scores of perceived physical appearance across all Ablon’s ratings done by an external examiner. However, in the women perceived physical appearance had a low, but significant, correlation with the external rating; this was the most evident in women with the highest Ablon’s index score, who had the lowest scores on self-perceived image. This contrasts with a previous study conducted in Australia, reflecting the body image concerns of Australian adults, where men and women were equally bothered by the appearance of NF1 visibility manifestations [12]. However, our findings are consistent with a study conducted in Norway where the appearance of NF1 was a major concern for women, whereas men expressed little concern about the visible manifestations of NF1 [13]. A study conducted in the US found that a high proportion of women with NF1 had appearance concerns, and were associated with low self-esteem and feelings of social isolation, although that study was done exclusively in females so there is no direct comparison to men [26]. Some of these differences may reflect how physical appearance if valued by different cultures and also by geographical issues. For example, in Australia the warmer weather and beach culture probably implies that men have more skin exposed and thus, may be more aware of the disease visibility than in a colder climate, such as Norway, where they can be covered much of the year. Additionally, there are methodological explanations as the Australian and Norwegian studies were qualitative in nature, so they may not be able to detect differences if they exist. Further work in larger cohorts of individuals with NF1 around the works will help understand how different cultures interact with gender and body image in NF1.

In our study, women with NF1 also reported worse mental health than men. While a cross-sectional study cannot address causation, it is likely that the effect on mental and emotional health is related in part to worse body image. Especially as we did not find significant differences in other markers of disease burden between genders, for example related to prevalence of optic glioma, spinal tumours, plexiform neurofibroma, pain interference scores, educational attainment, marital or employment status. Previous studies in NF1 have shown that disease visibility negatively affected emotional and mental health, physical symptoms, social functioning, and overall quality of life [11, 14, 27–29]. Kodra et al. found that approximately 40% of patients with NF1 reported feeling embarrassed about their skin condition and more than 20% worried about having scars [27]. Other studies have shown that people with NF1 appraised their own bodies more negatively than patients with psoriasis and anorexia nervosa [25]. Women with disabilities may have more limited role choices and models than do men, and are more likely to internalize societal rejection than men with disabilities [30]. This may also account for the impact of visible disease in women with NF1 compared to men.

We did not find significant differences in overall quality of life between women and men with NF1 when using generic measures such as the SF-36 or EQ-5D-5L. The scores on the SF-36 were significantly lower for men and women compared to Canadian norms, but differences between men and women may be too small to detect in this smaller sample. However, when using an NF1-specific measure, women had worse overall QoL, as well as worse scores in perceived physical appearance, anxiety, and emotional health. This discrepancy in results suggests that generic measures of QoL may not be sensitive enough to capture certain domains that are relevant for people living with NF1. This may also explain why generic measures such as the EQ-5D-5L and its associated VAS score showed relatively high mean scores in this cohort, as they probably cannot capture well the effects of disfigurement, stigma, and associated social impacts such as effects on employment and social relations. Recommendations for QoL measures in NF1 have been recently published and can help researchers to choose appropriate measures for future studies [31].

A limitation of our study was that we were only able to shed light on few aspects of the experience of people living with NF1. We only had the perceived physical appearance domain of the Peds-QL NF1 module to assess physical appearance, and we do acknowledge that there are specific body image and disfigurement measures that would provide better understanding of body image in NF1. In addition, we did not explore mechanisms to cope with visible lesions; for example, in other skin disorders people can use makeup, which may not help for prominent cutaneous neurofibromas. We also enrolled individuals followed at a tertiary academic centre in Canada, so our results may not be generalizable to other settings. For example, it is possible that people with milder forms of NF1 are followed in community settings, and in these individuals, NF1 may have less impact in QoL, with less evident gender differences.

## Conclusions

In summary, we found that women with NF1 experience worse QoL than men, with worse perceived physical appearance, anxiety and mental health. Because perceived physical appearance has a low correlation to disease visibility, as assessed by a clinician, healthcare providers should not make assumptions about the impact of cutaneous manifestations of NF1, and should inquire about body image, physical appearance concerns, and mental health, especially among female patients with NF1.

## Data Availability

The datasets used and/or analysed during the current study are available from the corresponding author on reasonable request.

## Abbreviations

NF1: Neurofibromatosis type 1
QoL: Quality of life
MPNST: Malignant peripheral nerve sheath tumour
SF-36: Short form 36
EQ-5D-5L: EuroQol-5 Dimentions-5 Levels
PROMIS: Patient-Reported Outcomes Measurements Information System
NIH: National Institutes of Health
